# Crystal structure of 3-[(2-acetyl­phen­oxy)carbon­yl]benzoic acid

**DOI:** 10.1107/S1600536814021904

**Published:** 2014-10-11

**Authors:** Mohammad Shoaib, Ismail Shah, Syed Wadood Ali Shah, Muhammad Nawaz Tahir, Shafi Ullah, Muhammad Ayaz

**Affiliations:** a Department of Pharmacy, University of Malakand, Khyber Pakhtunkhwa, Pakistan; b Department of Physics, University of Sargodha, Sargodha, Punjab, Pakistan

**Keywords:** crystal structure, 3-[(2-acetyl­phen­oxy)carbon­yl]benzoic acid, hydrogen bonding, 2′-hy­droxy­aceto­phenone, isopthaloyl chloride

## Abstract

In the title compound, C_16_H_12_O_5_, synthesized from isopthaloyl chloride and 2′-hy­droxy­aceto­phenone, the dihedral angle between the planes of the aromatic rings is 71.37 (9)°. In the crystal, carb­oxy­lic acid inversion dimers generate *R*_2_^2^(8) loops. The dimers are linked by C—H⋯O inter­actions, generating (101) sheets.

## Related literature   

For related structures, see: Derissen (1974 ▶); Tanimoto *et al.* (1973 ▶).
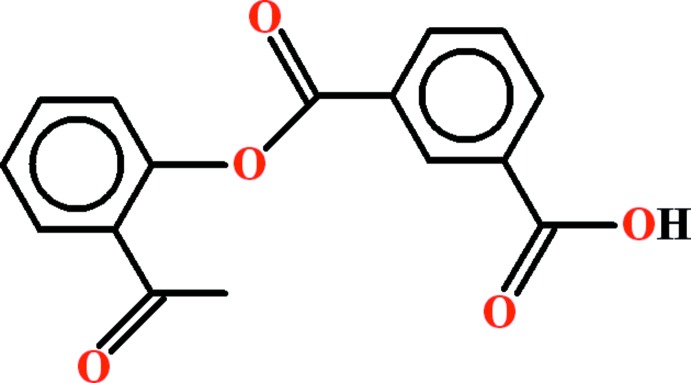


## Experimental   

### Crystal data  

C_16_H_12_O_5_
*M* *_r_* = 284.26
Monoclinic, 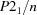

*a* = 13.5081 (10) Å
*b* = 7.4743 (6) Å
*c* = 13.9421 (11) Å
β = 106.671 (3)°
*V* = 1348.48 (18) Å^3^
*Z* = 4
Mo *K*α radiation
μ = 0.11 mm^−1^
*T* = 296 K
0.38 × 0.28 × 0.25 mm

### Data collection  

Bruker Kappa APEXII CCD diffractometer
Absorption correction: multi-scan (*SADABS*; Bruker, 2005 ▶) *T*_min_ = 0.963, *T*_max_ = 0.977
10280 measured reflections
2655 independent reflections
1971 reflections with *I* > 2σ(*I*)
*R*_int_ = 0.022

### Refinement  

*R*[*F* ^2^ > 2σ(*F* ^2^)] = 0.041
*wR*(*F* ^2^) = 0.115
*S* = 1.03
2655 reflections
192 parameters
H-atom parameters constrained
Δρ_max_ = 0.18 e Å^−3^
Δρ_min_ = −0.20 e Å^−3^

### 

Data collection: *APEX2* (Bruker, 2007 ▶); cell refinement: *SAINT* (Bruker, 2007 ▶); data reduction: *SAINT*; program(s) used to solve structure: *SHELXS97* (Sheldrick, 2008 ▶); program(s) used to refine structure: *SHELXL97* (Sheldrick, 2008 ▶); molecular graphics: *ORTEP-3 for Windows* (Farrugia, 2012 ▶) and *PLATON* (Spek, 2009 ▶); software used to prepare material for publication: *WinGX* (Farrugia, 2012 ▶) and *PLATON*.

## Supplementary Material

Crystal structure: contains datablock(s) global, I. DOI: 10.1107/S1600536814021904/hb7293sup1.cif

Structure factors: contains datablock(s) I. DOI: 10.1107/S1600536814021904/hb7293Isup2.hkl

Supporting information file. DOI: 10.1107/S1600536814021904/hb7293Isup3.cml

**Fig. 1.**
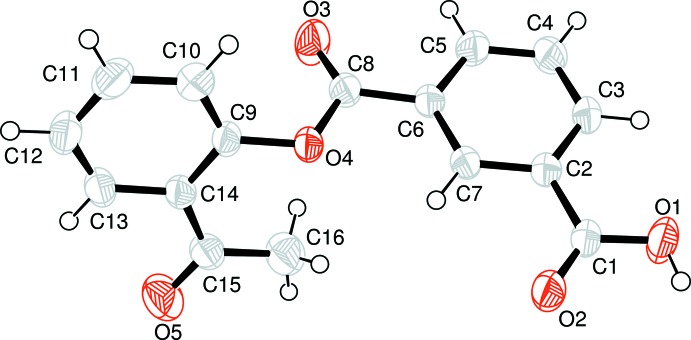
View of the title compound with displacement ellipsoids drawn at the 50% probability level.

. DOI: 10.1107/S1600536814021904/hb7293fig1.tif
View of the title compound with displacement ellipsoids drawn at the 50% probability level.

**Fig. 2.**
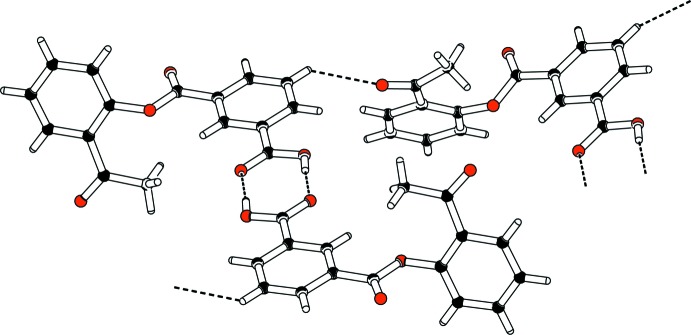
The partial packing (PLATON; Spek, 2009), which shows that molecules form dimers which are interlinked.

PLATON . DOI: 10.1107/S1600536814021904/hb7293fig2.tif
The partial packing (*PLATON*; Spek, 2009), which shows that mol­ecules form dimers which are inter­linked.

CCDC reference: 1027627

Additional supporting information:  crystallographic information; 3D view; checkCIF report

## Figures and Tables

**Table 1:** Hydrogen-bond geometry (, )

*D*H*A*	*D*H	H*A*	*D* *A*	*D*H*A*
O1H1O2^i^	0.82	1.84	2.6623(18)	175
C4H4O5^ii^	0.93	2.58	3.257(3)	130

Symmetry codes: (i) 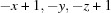; (ii) 
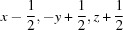
.

## supplementary crystallographic information

### S1. Comment

The title compound (I), (Fig. 1) has been synthesized for forming different metal complexes. The crystal structures of isophthalic acid and acetophenone have been published by (Derissen, 1974) and (Tanimoto, *et al.*, 1973) which are related to the title compound (I).

In (I) the group A (C1—C8/O1—O4) being like a part of isophthalic acid and benzene ring attached to it B (C9—C13) are almost planar with r. m. s. deviation of 0.0308 and 0.0034 Å, respectively. The dihedral angle between A/B is 71.98 (5)°. The acetaldehyde group C (O5/C15/C16) attached to ring B is of course planar. The dihedral angle between B/C is 9.56 (23)°. The molecules are dimerized due to coventional H-bondings of O—H···O type (Table 1, Fig. 2) forming *R*_2_^2^(8) loop. The dimers are further interlinked due to C—H···O bondings where C—H is of benzene containing carboxylate and O is of acetaldehyde group.

### S2. Experimental

Isopthaloyl chloride (25 mmol) and 2'-hydroxyacetophenone (35 mmol) were refluxed in the aquauos solution of pyridine for 30 min. The mixture was cooled to room temperature and added to a beaker containing 2 N HCl. The crushed ice was added and stirred vigorously. The precipitate formed were obtained though filteration. The column chromatography was done ethyl acetate:n-hexane (4:6) to obtain the pure product. Light yellow prisms were obtained after two days.

### S3. Refinement

All H atoms were geometrically placed [(O–H = 0.82 Å (hydroxyl), C–H = 0.93 Å (aromatic) and C–H = 0.96 Å (methyl) and refined as riding with with *U*_iso_(H) = *xU*_eq_(C, O), where *x* = 1.5 for hydroxy & methyl and *x* = 1.2 for aromatic H-atoms.

### Crystal data

**Table:**

C_16_H_12_O_5_	*F*(000) = 592
*M**_r_* = 284.26	*D*_x_ = 1.400 Mg m^−^^3^
Monoclinic, *P*2_1_/*n*	Mo *K*α radiation, λ = 0.71073 Å
*a* = 13.5081 (10) Å	Cell parameters from 1971 reflections
*b* = 7.4743 (6) Å	θ = 1.9–26.0°
*c* = 13.9421 (11) Å	µ = 0.11 mm^−^^1^
β = 106.671 (3)°	*T* = 296 K
*V* = 1348.48 (18) Å^3^	Prism, light yellow
*Z* = 4	0.38 × 0.28 × 0.25 mm

### Data collection

**Table:**

Bruker Kappa APEXII CCD diffractometer	2655 independent reflections
Radiation source: fine-focus sealed tube	1971 reflections with *I* > 2σ(*I*)
Graphite monochromator	*R*_int_ = 0.022
Detector resolution: 7.50 pixels mm^-1^	θ_max_ = 26.0°, θ_min_ = 1.9°
ω scans	*h* = −15→16
Absorption correction: multi-scan (*SADABS*; Bruker, 2005)	*k* = −6→9
*T*_min_ = 0.963, *T*_max_ = 0.977	*l* = −17→17
10280 measured reflections	

### Refinement

**Table:**

Refinement on *F*^2^	Primary atom site location: structure-invariant direct methods
Least-squares matrix: full	Secondary atom site location: difference Fourier map
*R*[*F*^2^ > 2σ(*F*^2^)] = 0.041	Hydrogen site location: inferred from neighbouring sites
*wR*(*F*^2^) = 0.115	H-atom parameters constrained
*S* = 1.03	*w* = 1/[σ^2^(*F*_o_^2^) + (0.0505*P*)^2^ + 0.4011*P*] where *P* = (*F*_o_^2^ + 2*F*_c_^2^)/3
2655 reflections	(Δ/σ)_max_ < 0.001
192 parameters	Δρ_max_ = 0.18 e Å^−^^3^
0 restraints	Δρ_min_ = −0.20 e Å^−^^3^

### Special details

**Table:**

Geometry. All e.s.d.'s (except the e.s.d. in the dihedral angle between two l.s. planes) are estimated using the full covariance matrix. The cell e.s.d.'s are taken into account individually in the estimation of e.s.d.'s in distances, angles and torsion angles; correlations between e.s.d.'s in cell parameters are only used when they are defined by crystal symmetry. An approximate (isotropic) treatment of cell e.s.d.'s is used for estimating e.s.d.'s involving l.s. planes.
Refinement. Refinement of *F*^2^ against ALL reflections. The weighted *R*-factor *wR* and goodness of fit *S* are based on *F*^2^, conventional *R*-factors *R* are based on *F*, with *F* set to zero for negative *F*^2^. The threshold expression of *F*^2^ > σ(*F*^2^) is used only for calculating *R*-factors(gt) *etc*. and is not relevant to the choice of reflections for refinement. *R*-factors based on *F*^2^ are statistically about twice as large as those based on *F*, and *R*-factors based on ALL data will be even larger.

### Fractional atomic coordinates and isotropic or equivalent isotropic displacement parameters (Å^2^)

**Table:**

	*x*	*y*	*z*	*U*_iso_*/*U*_eq_	
O1	0.36130 (10)	0.0311 (2)	0.47366 (9)	0.0704 (5)	
H1	0.4166	0.0276	0.5177	0.106*	
O2	0.46474 (9)	−0.0058 (2)	0.37694 (9)	0.0627 (4)	
O3	0.14695 (9)	−0.0066 (2)	−0.05814 (9)	0.0669 (4)	
O4	0.31564 (8)	−0.05274 (16)	0.01634 (7)	0.0411 (3)	
O5	0.48190 (13)	0.2965 (2)	−0.11562 (12)	0.0767 (5)	
C1	0.37753 (13)	0.0154 (2)	0.38694 (11)	0.0435 (4)	
C2	0.28422 (12)	0.0221 (2)	0.29912 (11)	0.0370 (4)	
C3	0.18668 (13)	0.0478 (2)	0.31086 (12)	0.0435 (4)	
H3	0.1791	0.0628	0.3746	0.052*	
C4	0.10086 (13)	0.0512 (3)	0.22840 (13)	0.0505 (5)	
H4	0.0355	0.0673	0.2366	0.061*	
C5	0.11211 (13)	0.0306 (3)	0.13359 (13)	0.0471 (4)	
H5	0.0542	0.0336	0.0780	0.056*	
C6	0.20966 (12)	0.0052 (2)	0.12066 (11)	0.0368 (4)	
C7	0.29593 (12)	0.0005 (2)	0.20355 (11)	0.0370 (4)	
H7	0.3613	−0.0169	0.1955	0.044*	
C8	0.21703 (12)	−0.0183 (2)	0.01684 (12)	0.0404 (4)	
C9	0.33430 (12)	−0.0900 (2)	−0.07617 (11)	0.0381 (4)	
C10	0.30562 (14)	−0.2564 (3)	−0.11818 (13)	0.0485 (4)	
H10	0.2692	−0.3350	−0.0892	0.058*	
C11	0.33147 (15)	−0.3054 (3)	−0.20375 (13)	0.0560 (5)	
H11	0.3132	−0.4178	−0.2319	0.067*	
C12	0.38433 (14)	−0.1876 (3)	−0.24715 (13)	0.0552 (5)	
H12	0.4012	−0.2199	−0.3049	0.066*	
C13	0.41194 (13)	−0.0225 (3)	−0.20493 (12)	0.0476 (5)	
H13	0.4474	0.0559	−0.2351	0.057*	
C14	0.38845 (12)	0.0318 (2)	−0.11770 (11)	0.0375 (4)	
C15	0.42394 (13)	0.2153 (2)	−0.07883 (12)	0.0456 (4)	
C16	0.38994 (17)	0.3003 (3)	0.00334 (16)	0.0614 (5)	
H16A	0.4174	0.4195	0.0148	0.092*	
H16B	0.4148	0.2311	0.0635	0.092*	
H16C	0.3158	0.3051	−0.0154	0.092*	

### Atomic displacement parameters (Å^2^)

**Table:**

	*U*^11^	*U*^22^	*U*^33^	*U*^12^	*U*^13^	*U*^23^
O1	0.0455 (8)	0.1379 (14)	0.0272 (6)	0.0069 (9)	0.0097 (5)	−0.0055 (7)
O2	0.0363 (7)	0.1189 (13)	0.0323 (6)	0.0023 (7)	0.0089 (5)	−0.0024 (7)
O3	0.0391 (7)	0.1245 (13)	0.0328 (7)	0.0139 (7)	0.0037 (5)	0.0015 (7)
O4	0.0325 (6)	0.0653 (8)	0.0264 (5)	−0.0005 (5)	0.0097 (4)	−0.0007 (5)
O5	0.0916 (11)	0.0748 (10)	0.0791 (10)	−0.0290 (9)	0.0495 (9)	−0.0073 (8)
C1	0.0401 (10)	0.0629 (12)	0.0286 (8)	−0.0013 (8)	0.0116 (7)	−0.0012 (7)
C2	0.0356 (9)	0.0454 (10)	0.0305 (8)	−0.0018 (7)	0.0104 (6)	0.0008 (6)
C3	0.0430 (10)	0.0594 (11)	0.0321 (8)	−0.0023 (8)	0.0169 (7)	−0.0031 (7)
C4	0.0338 (9)	0.0763 (13)	0.0449 (10)	0.0017 (9)	0.0171 (8)	−0.0054 (9)
C5	0.0329 (9)	0.0706 (13)	0.0360 (9)	0.0009 (8)	0.0071 (7)	−0.0027 (8)
C6	0.0324 (8)	0.0483 (10)	0.0300 (8)	−0.0011 (7)	0.0097 (6)	0.0008 (7)
C7	0.0313 (8)	0.0500 (10)	0.0312 (8)	−0.0018 (7)	0.0113 (6)	0.0011 (7)
C8	0.0317 (8)	0.0574 (11)	0.0311 (8)	0.0010 (7)	0.0074 (7)	0.0027 (7)
C9	0.0317 (8)	0.0567 (11)	0.0256 (7)	0.0028 (7)	0.0077 (6)	−0.0007 (7)
C10	0.0474 (10)	0.0557 (11)	0.0418 (9)	−0.0069 (9)	0.0118 (8)	−0.0027 (8)
C11	0.0544 (11)	0.0617 (13)	0.0480 (10)	−0.0025 (9)	0.0082 (9)	−0.0173 (9)
C12	0.0499 (11)	0.0804 (15)	0.0376 (9)	0.0033 (10)	0.0161 (8)	−0.0135 (9)
C13	0.0411 (9)	0.0692 (13)	0.0362 (9)	0.0021 (8)	0.0172 (7)	0.0005 (8)
C14	0.0296 (8)	0.0522 (10)	0.0307 (8)	0.0036 (7)	0.0087 (6)	0.0012 (7)
C15	0.0404 (9)	0.0555 (11)	0.0419 (9)	−0.0006 (8)	0.0135 (8)	0.0047 (8)
C16	0.0714 (14)	0.0531 (12)	0.0682 (13)	−0.0077 (10)	0.0335 (11)	−0.0133 (10)

### Geometric parameters (Å, º)

**Table:**

O1—C1	1.2946 (19)	C6—C8	1.489 (2)
O1—H1	0.8200	C7—H7	0.9300
O2—C1	1.236 (2)	C9—C10	1.382 (2)
O3—C8	1.1953 (19)	C9—C14	1.394 (2)
O4—C8	1.3587 (19)	C10—C11	1.385 (2)
O4—C9	1.4111 (17)	C10—H10	0.9300
O5—C15	1.215 (2)	C11—C12	1.379 (3)
C1—C2	1.485 (2)	C11—H11	0.9300
C2—C3	1.387 (2)	C12—C13	1.372 (3)
C2—C7	1.395 (2)	C12—H12	0.9300
C3—C4	1.379 (2)	C13—C14	1.402 (2)
C3—H3	0.9300	C13—H13	0.9300
C4—C5	1.382 (2)	C14—C15	1.502 (2)
C4—H4	0.9300	C15—C16	1.493 (2)
C5—C6	1.393 (2)	C16—H16A	0.9600
C5—H5	0.9300	C16—H16B	0.9600
C6—C7	1.386 (2)	C16—H16C	0.9600
			
C1—O1—H1	109.5	C10—C9—O4	117.60 (14)
C8—O4—C9	118.33 (12)	C14—C9—O4	120.23 (15)
O2—C1—O1	122.65 (15)	C9—C10—C11	119.60 (17)
O2—C1—C2	121.50 (14)	C9—C10—H10	120.2
O1—C1—C2	115.84 (14)	C11—C10—H10	120.2
C3—C2—C7	120.03 (15)	C12—C11—C10	119.96 (18)
C3—C2—C1	121.19 (14)	C12—C11—H11	120.0
C7—C2—C1	118.78 (14)	C10—C11—H11	120.0
C4—C3—C2	120.27 (15)	C13—C12—C11	119.85 (16)
C4—C3—H3	119.9	C13—C12—H12	120.1
C2—C3—H3	119.9	C11—C12—H12	120.1
C3—C4—C5	119.91 (15)	C12—C13—C14	122.08 (17)
C3—C4—H4	120.0	C12—C13—H13	119.0
C5—C4—H4	120.0	C14—C13—H13	119.0
C4—C5—C6	120.44 (15)	C9—C14—C13	116.61 (16)
C4—C5—H5	119.8	C9—C14—C15	126.71 (14)
C6—C5—H5	119.8	C13—C14—C15	116.68 (15)
C7—C6—C5	119.71 (14)	O5—C15—C16	119.23 (18)
C7—C6—C8	122.18 (14)	O5—C15—C14	118.82 (16)
C5—C6—C8	118.11 (14)	C16—C15—C14	121.95 (15)
C6—C7—C2	119.65 (15)	C15—C16—H16A	109.5
C6—C7—H7	120.2	C15—C16—H16B	109.5
C2—C7—H7	120.2	H16A—C16—H16B	109.5
O3—C8—O4	122.75 (15)	C15—C16—H16C	109.5
O3—C8—C6	125.75 (15)	H16A—C16—H16C	109.5
O4—C8—C6	111.49 (13)	H16B—C16—H16C	109.5
C10—C9—C14	121.90 (14)		
			
O2—C1—C2—C3	−179.32 (18)	C5—C6—C8—O4	−176.17 (15)
O1—C1—C2—C3	1.4 (3)	C8—O4—C9—C10	−74.90 (19)
O2—C1—C2—C7	1.2 (3)	C8—O4—C9—C14	110.95 (17)
O1—C1—C2—C7	−178.08 (17)	C14—C9—C10—C11	0.3 (3)
C7—C2—C3—C4	0.4 (3)	O4—C9—C10—C11	−173.72 (15)
C1—C2—C3—C4	−179.14 (17)	C9—C10—C11—C12	−0.8 (3)
C2—C3—C4—C5	−0.6 (3)	C10—C11—C12—C13	0.5 (3)
C3—C4—C5—C6	0.4 (3)	C11—C12—C13—C14	0.2 (3)
C4—C5—C6—C7	0.0 (3)	C10—C9—C14—C13	0.4 (2)
C4—C5—C6—C8	179.31 (17)	O4—C9—C14—C13	174.31 (13)
C5—C6—C7—C2	−0.3 (3)	C10—C9—C14—C15	−179.79 (16)
C8—C6—C7—C2	−179.53 (15)	O4—C9—C14—C15	−5.9 (2)
C3—C2—C7—C6	0.1 (2)	C12—C13—C14—C9	−0.7 (2)
C1—C2—C7—C6	179.60 (15)	C12—C13—C14—C15	179.48 (16)
C9—O4—C8—O3	−5.1 (3)	C9—C14—C15—O5	170.50 (17)
C9—O4—C8—C6	175.91 (14)	C13—C14—C15—O5	−9.7 (2)
C7—C6—C8—O3	−175.87 (18)	C9—C14—C15—C16	−9.6 (3)
C5—C6—C8—O3	4.9 (3)	C13—C14—C15—C16	170.19 (17)
C7—C6—C8—O4	3.1 (2)		

### Hydrogen-bond geometry (Å, º)

**Table:**

*D*—H···*A*	*D*—H	H···*A*	*D*···*A*	*D*—H···*A*
O1—H1···O2^i^	0.82	1.84	2.6623 (18)	175
C4—H4···O5^ii^	0.93	2.58	3.257 (3)	130

Symmetry codes: (i) −*x*+1, −*y*, −*z*+1; (ii) *x*−1/2, −*y*+1/2, *z*+1/2.
