# Supplementary figures and images for: Crystal structure of 3-[(2-acetyl­phen­oxy)carbon­yl]benzoic acid
Source: Acta Crystallogr Sect E Struct Rep Online. 2014 Oct 11;70(Pt 11):o1153. doi: 10.1107/S1600536814021904 (PMC4257273; doi:10.1107/S1600536814021904)

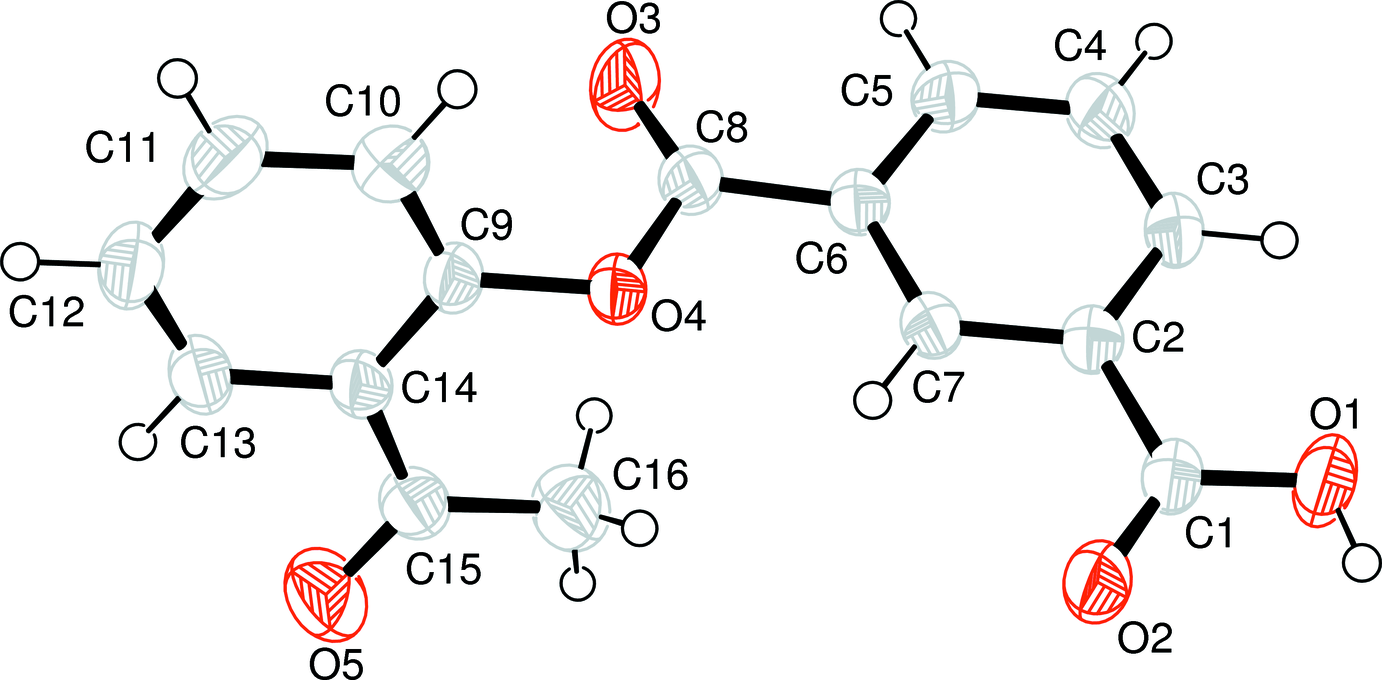

Supplement: Supplementary file 4 [file e-70-o1153-fig1.tif]

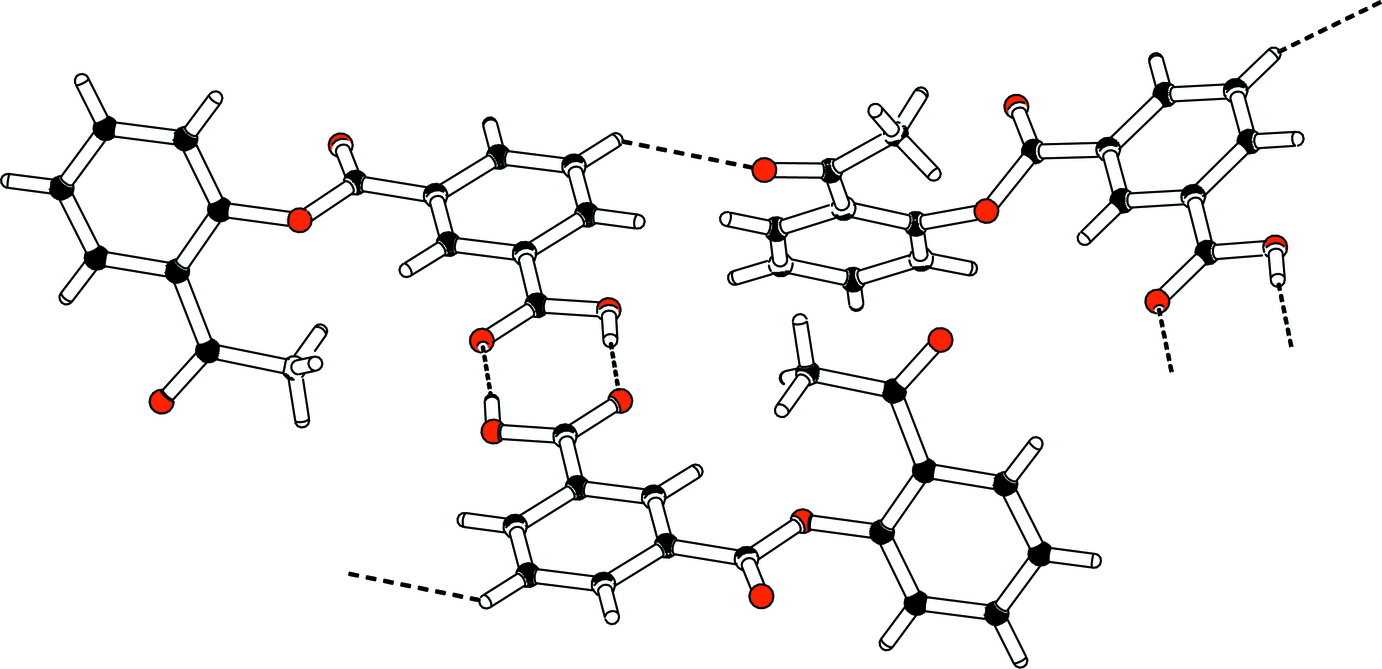

Supplement: Supplementary file 5 [file e-70-o1153-fig2.tif]
